# Effect of electrode placement on speed of response to ECT

**DOI:** 10.1192/j.eurpsy.2021.83

**Published:** 2021-08-13

**Authors:** D. Mcloughlin, C. Fox

**Affiliations:** Psychiatry, Trinity College Dublin, Dublin, Ireland

**Keywords:** response, Electroconvulsive therapy, Depression, remission

## Abstract

**Objective:**

Electroconvulsive therapy (ECT) can be rapidly effective in treating severe depression. Right unilateral (RUL) or bitemporal (BT) electrode placement may affect the speed of ECT effectiveness although our current understanding of demographic and clinical factors for predicting predict speed of response and remission with ECT is limited. We investigated differences in improvement speed and also time to achieving response and remission criteria between brief-pulse moderate-dose (1.5 x seizure threshold) BT ECT and high-dose (6 x seizure threshold) RUL ECT. Additionally, we explored the influence of demographic and clinical characteristics.

**Methods:**

Se analysed weekly 24-item Hamilton Depression Rating Scale scores obtained from severely depressed patients participating in the EFFECT-Dep trial (ISRCTN23577151). Improvement speeds in patients treated randomly with a course of either BT (n = 69) or RUL ECT (n = 69) were compared using independent sample t-tests. Weekly proportions of responders and remitters were compared using chi-square tests. Cox regression analyses were used to explore predictors of speed to achieve response and remission status.

**Results:**

Se found no differences between RUL and BT ECT in speed of improvement or time to achieve response or remission. Exploratory analyses indicated that a wide variety of demographic and clinical features did not serve to predict speed of response and remission to ECT.

**Conclusion:**

Electrode placement did not substantially influence speed of improvement, response and remission with twice-weekly brief-pulse ECT. Minimising the cognitive side-effects of ECT may be of more relevance when choosing between BT and RUL electrode placement for ECT.

**Disclosure:**

Declan M. McLoughlin has received speaker’s honoraria from Mecta and Otsuka and an honorarium from Janssen for participating in an esketamine advisory board meeting. The other author reports no conflicts of interest. This work was supported by awards from

